# Atlas and Epitomè of Gynecology

**Published:** 1900-11

**Authors:** 


Atlas and Epitomè of Gynecology. By Dr. Oskar Schaefer, Privatdocent of Obstetrics and Gynecology in the University of Heidelberg. Edited by Richard C. Norris, A M., M. D., Gynecologist to the Methodist Episcopal Hospital and to the Philadelphia Hospital; Lecturer on Clinical and Operative Obstetrics, Medical Department University of Pennsylvania. 207 colored illustrations on 90 plates and 62 text illustrations. Philadelphia: W. B. Saunders & Co. 1900.

Atlas and Epitomè of Diseases Caused by Accidents. By Dr. Ed. Golcbiewski, of Berlin. Authorized Translation from the German, with Editorial Notes and Additions, by Pearce Bailey, M. D., Consulting Neurologist to St. Luke's Hospital, New York; Assistant in Neurology, Columbia University; Author of "Accidents and Injury; their Relations to Diseases of the Nervous System." Forty colored plates and 143 text illustrations. Philadelphia: W. B. Saunders & Co. 1900.

The man who writes a good book is an educator; the man who prints the book and gives it to the public is a benefactor. When Saunders first announced the publication of the Atlas Series, of which these two volumes are part, it was said that the volume would be as perfect as it was possible to make them. How well this has been done we all know, yet we cannot help admiring the exquisite book-work shown in the production of these volumes. They show the highest type of serviceable bookmaking. In scope they, as a class, stand midway between the dear old quiz compend of student days and the heavier text-books.

In the gynecology the illustrations are particularly valuable, being reproductions of water-color paintings made from fresh specimens by the best German artists. So well has this work been done that the student obtains from the plates a very excellent mental picture of the pathologic changes wrought by disease of the pelvic organs, which he could not get from any wordy description however well done, however clearly stated. There is added to this the kind of knowledge one absorbs from a study of the specimens or a visit to the clinic. The handling of the subject has been most admirable and thorough.

Anomalies of formation and arrested development begin the book, followed by changes of shape and position of the pelvic organs. Then inflammatory and nutritional disturbances are taken up, and considered in a manner almost wholly different from what we are used to in this part of the globe. The German thoroughness is everywhere apparent, especially in the closing chapters dealing with injuries and their consequences and new growths, which takes in the very extensive subject of tumors. The closing pages of the book are devoted to a very valuable therapeutic table which is such a splendid guide to every-day gynecological work.

In reviewing, or even casually examining the Atlas of Diseases caused by Accidents, one is forcibly reminded of the shortcomings of American medical education, which in the majority of colleges almost wholly ignore, or at best make incidental and passing references to the subject which this volume so clearly sets forth. This was referred to in the JOURNAL when Saunders's Atlas of Legal Medicine was reviewed, and again in an editorial article. In these days of negligence cases, when the number of lawyers who make a specialty of damage suits for personal injury is on the increase, when one half the jury cases in this country are tried for personal injuries, and when whole armies of factory employees are insured against accident by insurance companies by the blanket policy system, information, such as is set forth in this book is not only of great assistance to a physician or a surgeon, but is an absolute necessity.

The work, while primarily a medical book, is designed to be of aid to the legal fraternity and to the accident insurance companies. The introduction by Dr. Bailey, the editor, is intensely interesting. He shows in concise, temperate language, the difference between the German and American systems of law making to regulate accident indemnity. The German system which has been in force since 1884 permits of increase or decrease in indemnity as the patient gets worse or better. In this country, a jury, after hearing a raft of "expert"

evidence by medical men who have been paid to swear to certain specific statements, gives a verdict for a fixed sum which settles the case.

To the general practitioner this atlas is the most valuable of the series, for it tells him things he does not know and cannot know, for he has never been taught them. Here are shown him in splendid, life-like coloring the result of injuries and operations; statistics are referred to at sufficient length to permit him to talk intelligently of the probable duration of disability following accident, and he is given a very good general working knowledge of what the amount of disability is and will be, all other things being equal. Dr. Bailey has added a great deal of American information to the translation, and there is no excuse for a medical man making an addle-pated exhibition of himself on the witness stand in an ordinary, everyday damage suit. The Atlas of Diseases Caused by Accident should have the largest sale of any of the Saunders' series, and a copy should be presented to the faculty of every medical college in the country which is ignoring the subject, to show them how they are not equipping medical students.

